# *DAO2* Modulates Gamma-Aminobutyric Acid Accumulation in Germinating Brown Rice

**DOI:** 10.3390/foods15213847

**Published:** 2026-10-28

**Authors:** Pingli Chen, Xudong Liu, Qing Liu, Chen Li, Bingrui Sun, Hang Yu, Liqun Jiang, Xingxue Mao, Jing Zhang, Shuwei Lv, Yanfei Zhou, Yilong Yuan, Shahzad Amir Naveed, Muhammad Ramzan Khan, Jianxiong Li

**Affiliations:** 1 Rice Research Institute, Guangdong Academy of Agricultural Sciences, Guangzhou 510640, China; 2 Key Laboratory of Genetics and Breeding of High Quality Rice in Southern China (Co-Construction by Ministry and Province), Ministry of Agriculture and Rural Affairs, Guangzhou 510640, China; 3 Guangdong Key Laboratory of Rice Science and Technology, Guangzhou 510640, China; 4 Guangdong Rice Engineering Laboratory, Guangzhou 510640, China; 5 Wuhan Qingfa Hesheng Seed Co., Ltd., Wuhan 430070, China; 6 National Institute for Genomics and Advanced Biotechnology (NIGAB), National Agricultural Research Center (NARC), Park Road, Islamabad 45500, Pakistan

**Keywords:** rice, gamma-aminobutyric acid, germinated brown rice, diamine oxidase, giant embryo, molecular mechanism

## Abstract

Gamma-aminobutyric acid (GABA) is a functional non-protein amino acid that regulates plant stress responses and signaling, while offering antihypertensive and hepatorenal benefits in humans. However, the genetic and molecular networks governing GABA accumulation during brown rice germination remain poorly understood. Here, we engineered *DAO2* knockout rice lines using CRISPR/Cas9. Mutant brown rice exhibited significantly lower GABA levels alongside a marked accumulation of glutamate (Glu), glutamine (Gln), arginine (Arg), and proline (Pro) compared to the wild type (WT). Spatiotemporal profiling across seven germination intervals revealed that GABA titers peaked at 24 h in both the *DAO2-1* mutant (24.24 mg/100 g) and WT (24.95 mg/100 g) before stabilizing. Notably, Glu levels were suppressed in *DAO2-1* relative to WT during germination, whereas Pro showed the inverse trend. Transcriptomic analysis indicated substantial temporal variations in GABA metabolic gene expression between the two genotypes. The differences in expression level of *OsGAD* and *OsGABA-T* in *DAO2-1* likely accounts for its diminished GABA capacity during early germination. These insights clarify the role of *DAO2* within the regulatory network of rice GABA metabolism, providing a biochemical blueprint for optimized processing and the targeted breeding of GABA-enriched functional rice varieties.

## 1. Introduction

Gamma-aminobutyric acid (GABA) is a ubiquitous non-protein amino acid. In humans and animals, it acts as a primary inhibitory neurotransmitter crucial for neurological homeostasis, whereas in plants, it mediates defense responses, growth, and maturation [1,2]. Guo et al. reviewed the roles of the synthesis and metabolism pathways of GABA in regulating primary metabolism of plants, redistributing carbon and nitrogen resources, reducing the accumulation of reactive oxygen species, and enhancing the tolerance of plants to oxidative stress [3]. Xu et al. explored the emerging network of GABA’s interactions with other signaling pathways, emphasizing its involvement in environmental sensing and internal regulatory mechanisms through hormones and reactive oxygen species [4]. The study positioned GABA as a metabolic signal integrator in plant physiological processes [4]. In human physiology, GABA drives cerebral energy metabolism by reducing blood ammonia, lowering blood pressure, and stabilizing neurological function [5]. The development of ligands for the binding sites of human GABA receptors, as well as the prospects of GABA production and application, were discussed [6].

The plant GABA metabolic network primarily integrates the GABA shunt and the polyamine degradation pathway [7]. In the GABA shunt, L-glutamate decarboxylase (GAD) directly converts L-glutamate (Glu) into GABA [8,9]. GAD is the rate-limiting enzyme governing biosynthesis and is encoded by a multi-gene family in rice, displaying pronounced tissue- and stage-specific expression profiles [10]. To date, five plant GAD genes (*OsGAD1* to *OsGAD5*) have been characterized [10]. Alternatively, the polyamine degradation pathway yields GABA from polyamines (PAs) through sequential enzymatic steps. PAs are processed into 4-aminobutyraldehyde by diamine oxidase (DAO) and polyamine oxidase (PAO), the rate-limiting enzymes of this bypass [7], and are subsequently converted to GABA via pyrroline dehydrogenase (PDH) and betaine aldehyde dehydrogenase (BADH2). While plant *DAO* variants are extensively characterized in *Arabidopsis thaliana*, only a single *DAO* locus has been cloned in rice [11]. Conversely, the rice genome harbors seven PAO-encoding genes (*OsPAO1* to *OsPAO7*) distributed across different chromosomes [12]. For catabolism, GABA is mitochondrially degraded into succinic acid by GABA transaminase (GABA-T) and succinic semialdehyde dehydrogenase (SSADH) to enter the tricarboxylic acid (TCA) cycle. Within this cycle, ⍺-ketoglutarate (⍺-KG) is converted back to Glu, preserving the sub-cellular GABA/Glu equilibrium [13]. So far, four rice OsGABA-T genes (OsGABA-T1 to OsGABA-T4) have been identified [14,15,16]. Additionally, alanine aminotransferase (AlaAT) mediates the reversible transamination of pyruvate and glutamate to alanine and *α*-KG, balancing intracellular amino acid pools during development and stress [17,18]. Five homologous AlaAT genes (*OsAlaAT1* to *OsAlaAT5*) are present in rice, with *OsAlaAT1* showing the strongest induction under hypoxic stress [19].

Due to its therapeutic potential, interest in GABA-fortified functional foods has escalated [20]. Germinated brown rice (GBR), retaining the bran layer and a 1–2 mm sprout, serves as an ideal vehicle [7]. The final GABA yield depends heavily on processing parameters; modifying the soaking, germination, drying, or environmental stress conditions alters accumulation dynamics. Germination triggers endogenous GAD activity to deaminate Glu into GABA, a process supported by proteolysis that continuously replenishes the free Glu pool [21]. Consequently, GABA levels in GBR typically range from 127.7 to 248.9 μg/g, roughly tripling that of unpolished grains (38.9 to 77.1 μg/g) [22]. Researchers have produced GABA-rich brown rice products by combining the biological metabolic pathway of GABA with traditional plant enrichment techniques [23,24,25,26,27]. For instance, Komatsuzaki et al. reported a GABA content of 24.9 mg/100 g after soaking brown rice for 3 h followed by a 21-h incubation at 35 °C [28]. Following draining, high-temperature sterilization preserved and boosted GABA retention by approximately 5% relative to alternative drying methods, matching observations by Young-Su Youn et al. [29]. Soaking duration also exerts a strong influence: Charoenthaikij et al. observed a rise from 327.0 μg/g to 670.0 μg/g when extending the steeping from 24 to 48 h [30]. Conversely, Karladee et al. noted a biphasic trend where GABA increased from 39.6 μg/g at 12 h to 178.7 μg/g at 24 h, before dropping to 99.1 μg/g at 36 h and 13.6 μg/g at 48 h [31]. Komatsuzaki et al. attained peak accumulation (422.0 μg/g) after 22 h of germination, further demonstrating that soaking in exogenous Glu or lactic acid solutions enhanced concentrations to 203.2 and 222.5 μg/g, respectively, compared to a distilled water control (146.7 μg/g) [28]. Temporal dynamics vary among studies [32]; Karladee et al. reported a rapid spike from 39.6 μg/g (0 h) to 1004.0 μg/g at 12 h, maxing out at 1787.0 μg/g at 24 h [31], while Wu et al. recorded a steady, gradual climb from 93.5 μg/g at 18 h to 192.1 μg/g at 72 h [1]. Collectively, these findings emphasize that adjusting temperature, duration, and soaking additives can fine-tune GABA production.

Giant embryo (GE) rice varieties represent another high-GABA functional matrix [7], featuring embryos 2- to 3-fold larger than conventional cultivars. The *GE* gene encodes a cytochrome P450 monooxygenase that acts as a negative regulator of embryo size [7]; its loss-of-function expands the embryo at the expense of the endosperm, whereas its overexpression accelerates endosperm cell proliferation, yielding a small-embryo/large-endosperm phenotype [33]. Currently, multiple giant embryo alleles have been discovered, such as *ge-1* to *ge-10*, *ge^s^*, and *ge^t^*, etc. [33,34]. In our previous work using CRISPR/Cas9 multi-target editing, we generated three distinct *ge* mutant lines [7]. Transcriptomic screening revealed a significant up-regulation of *DAO2* (*LOC_Os04g20164*) within GABA-rich mutant grains. Although *DAO2* is known to encode an amine oxidase precursor linked to polyamine degradation, its exact function within the broader GABA metabolic framework remains uncharacterized.

This study investigates the metabolic profiles and corresponding gene expression shifts in CRISPR-derived *DAO2* rice mutants across sequential germination stages. By evaluating these temporal variations, we clarify the preliminary contribution of *DAO2* to GABA enrichment, establishing a genetic framework for breeding high-GABA rice cultivars and optimizing functional food production.

## 2. Experimental Methods

### 2.1. Experimental Materials and Cultivation

The conventional *japonica* rice cultivar Zhonghua 11 (ZH11), *DAO2* knockout lines, and the previously established giant embryo mutant *ge-1* [7] were cultivated during the early and late seasons of 2025 at the experimental field in Guangzhou, China. Sowing and transplantation schedules were closely matched to local seasonal conditions: early season sowing occurred on March 9, and late season sowing on July 23. Each line was planted in a three-row plot with 12 plants per row, utilizing a plant spacing of 16.5 cm and a row spacing of 26 cm. Standard irrigation and fertilization protocols were maintained throughout development. To minimize edge effects, the central 10 plants of the middle row were harvested individually at maturity, air-dried, and stored at 4 °C prior to analysis.

### 2.2. CRISPR/Cas9 Targeting DAO2 (LOC_Os04g20164) Vector Construction and Plant Transformation

A dual-target CRISPR/Cas9 recombinant vector, designated CRISPR-DAO2, was constructed using the pRGEB32 backbone following established homologous recombination protocols as reported previously [35]. Two specific gRNA targets were selected within the *DAO2* locus: *DAO2*-gRNA1 (5′-GTGGTACTTCAAGACGTACA-3′) and *DAO2*-gRNA2 (5′-CGTCGACTGGGAGTTCCAGA-3′). The fully assembled construct was introduced into rice variety ZH11 via *Agrobacterium tumefaciens* EHA105-mediated transformation to generate T_0_ transgenic lines. The methods, steps and medium formulations for genetic transformation were mainly based on the transformation systems established by previous researchers [36,37]. Positive transformants and specific mutations were identified and confirmed by sequencing using the target-specific primers DAO2.1-F/R for target 1 and DAO2.2-F/R for target 2. The primers are detailed in Table 1.

### 2.3. Brown Rice Germination Mechanics

Mature grains from ZH11 and the *DAO2* mutants were dehulled using a TR 200 dehuller (Kett, Tokyo, Japan) to obtain unpolished brown rice. Sound, uniform seeds were selected and surface-sterilized with 75% ethanol for 1.5 min, followed by five consecutive rinses in sterile water. The seeds were pre-soaked in sterile water at 28 °C for 24 h, with the water refreshed every 6 h to maintain hygiene. For the germination assay, 12 discrete replicates per line (each comprising 30 intact grains) were arranged in sterile Petri dishes, moistened with sterile water, and incubated in the dark at 28 °C. Grains showing bacterial contamination or developmental arrest were discarded immediately to preserve culture integrity. Germinating samples were harvested at seven sequential intervals (8, 12, 18, 24, 30, 36, and 40 h) for downstream metabolite analysis and gene expression analysis, and stored immediately at −80 °C.

### 2.4. Metabolite Extraction and Quantification

Ungerminated and germinated brown rice grains were pulverized in liquid nitrogen using a cryogenic grinder Alpha 1-4 LSCLSCbasic (Martin Christ, Osterode, Germany) to pass through an 80-mesh sieve. Ground tissues (0.250 ± 0.001 g) were weighed precisely on an analytical balance, instantly stabilized in liquid nitrogen, and extracted in 6 mL of 0.1 mol/L aqueous HCl via ultrasonication at 35 °C for 60 min. A 500 µL aliquot of the extract was transferred to a clean tube, combined with 500 µL of 10% aqueous sulfosalicylic acid, and incubated at 4 °C for 1 h to precipitate proteins. After centrifugation at 12,000 rpm for 45 min at 4 °C, the supernatant was collected, the sample was filtered through a 0.22 μm microporous filter membrane (with a pore size of 0.22 μm), and it was stored in the sample vial. Quantitative detection of GABA, glutamate (Glu), glutamine (Gln), arginine (Arg), and proline (Pro) contents was performed using ultra-performance liquid chromatography–tandem mass spectrometry (UPLC-MS/MS) and evaluated across three biological replicates. This study was analyzed using LC-30AD UPLC/LCMS-8050 (MS/MS) (Shimadzu Corporation, Kyoto, Japan). The liquid phase conditions mainly included: the chromatographic column selected was ACQUITY UPLC BEH C18 (Waters, MA, USA) (2.1 × 100 mm Column, 1.7 um); the mobile phase A was ultra-pure water (with 0.1% acetic acid) and the mobile phase B was acetonitrile; the elution gradient: 0 min was A 95% (water phase/organic phase, *v*/*v*), 1 min was A 95% (*v*/*v*), 6 min was A 10% (*v*/*v*), 7.5 min was A 10% (*v*/*v*), 7.6 min was A 95% (*v*/*v*), and 12.5 min was A 95% (*v*/*v*); the flow rate was 0.3 mL/min, the injection volume was 2 μL, and the column temperature was 40 °C. The mass spectrometry conditions mainly included: ion source: ESI; positive ion mode; multiple reaction monitoring (MRM) for scanning; interface temperature: 300 °C; de-solvation temperature: 526 °C; de-solvation line temperature: 250 °C; heating block temperature: 400 °C; nebulizing gas flow rate: 3 L/min; heating gas and drying gas were both 10 L/min. Each substance ion pair was scanned and detected according to the optimized de-clustering potential (DP) and collision energy (CE).

### 2.5. RNA Isolation and RT-qPCR

Total RNA was isolated from pooled samples of 20 germinated grains per time point (8, 12, 18, 24, 30, 36, and 40 h; three biological replicates each) using Trizol reagent (Invitrogen, Carlsbad, CA, USA). First-strand cDNA was synthesized from 2 µg of total RNA using a PrimeScript™ RT Reagent Kit (Takara Bio Inc., Kusatsu, Japan), and the resulting cDNA products were diluted 20-fold for quantitative analysis. RT-qPCR was performed on three technical replicates using a SYBR Premix Ex Taq™ II Kit (Takara Bio Inc.) in a total reaction volume of 5 µL. The amplification program consisted of initial denaturation at 95 °C for 5 min, followed by 45 cycles of 95 °C for 15 s, 60 °C for 15 s, and 72 °C for 20 s. The rice *β-Actin* gene served as the internal reference control, and relative expression levels were calculated using the comparative 2^−^^ΔΔCT^ method. The specific primers used for expression analysis are detailed in Table 1.

### 2.6. Data Statistical Analysis

Statistical evaluation was conducted via two-tailed Student’s *t*-tests using Excel 2010 software. Significant differences between the mutant lines and the wild-type control are indicated by asterisks, where asterisks * and ** denote *p* < 0.05 and *p* < 0.01, respectively. All phenotypic measurements are expressed as mean ± standard deviation (SD).

## 3. Results

### 3.1. Generation and Characterization of DAO2 Mutants

**Figure 1:**
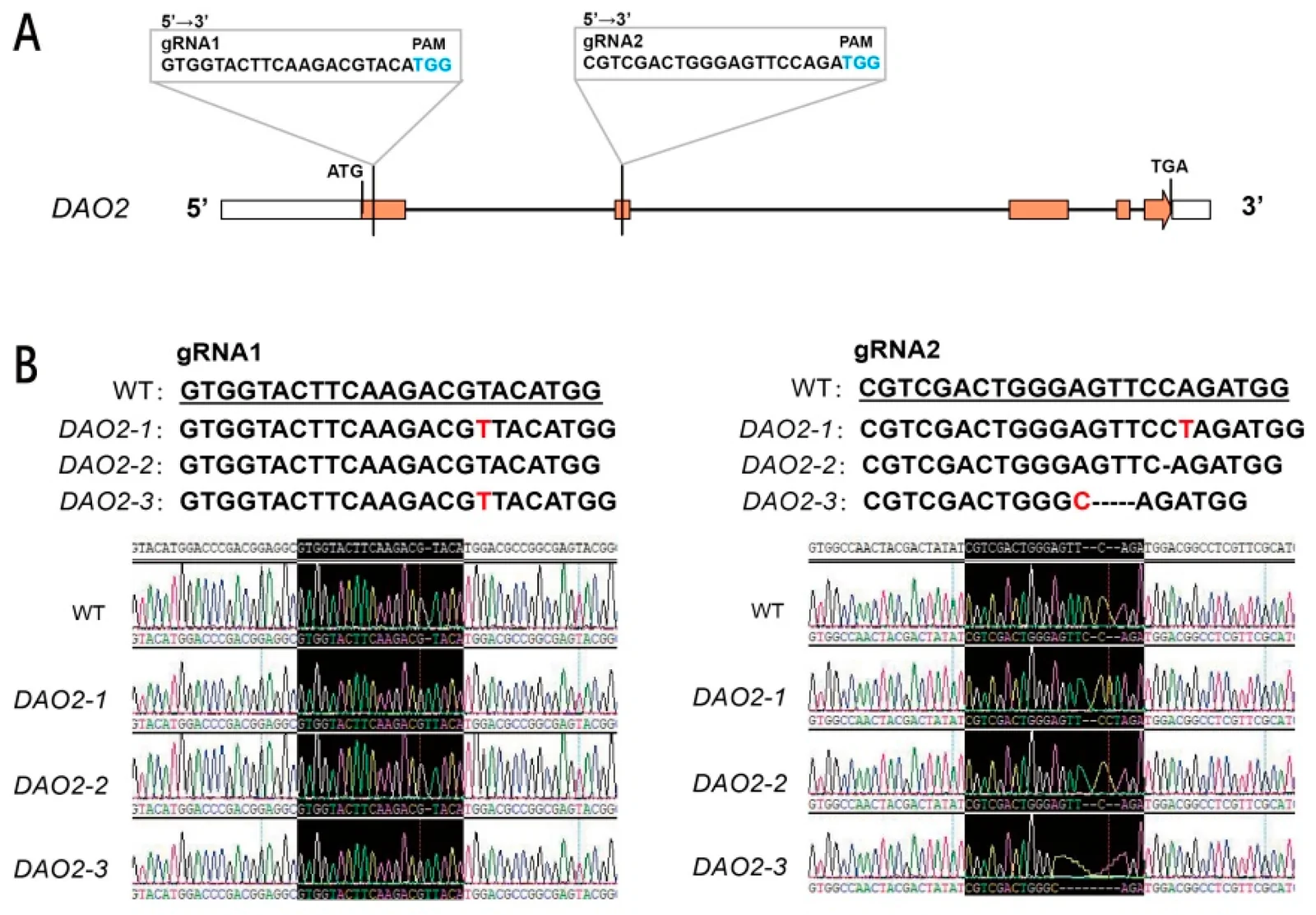
Generation and validation of *DAO2* mutants. (**A**) Schematic representation of the two sgRNA target sites engineered into the *DAO2* locus via the CRISPR/Cas9 multi-target vector. The protospacer adjacent motif (PAM) sequence is highlighted in blue. (**B**) Alignment of target site sequences from the homozygous mutant lines (*DAO2-1*, *DAO2-2*, and *DAO2-3*) against the WT control (ZH11).

To investigate whether *DAO2* disruption affects GABA accumulation in mature and germinated brown rice, we targeted the first and second exons of *DAO2* in the ZH11 background using a dual-target CRISPR/Cas9 editing strategy (Figure 1A). Hygromycin resistance screening and sequencing of 48 T_0_ transgenic lines identified 33 mutants and 15 wild-type (WT) segregants. Genotypic screening of subsequent generations using the specific primers DAO2.1-F/R and DAO2.2-F/R successfully isolated two independent T_2_ lines harboring homozygous mutations at both target sites. The three CRISPR mutant lines (*DAO2-1*, *DAO2-2*, and *DAO2-3*) were able to grow and mature normally under normal conditions. The sequence configurations for the selected mutant lines (*DAO2-1*, *DAO2-2*, and *DAO2-3*) are detailed in Figure 1B.

Sequencing confirmed that *DAO2-1* carries a single-base (T) insertion at position +130 bp of the first target site, causing a frameshift that alters the codon from TAC (Tyrosine) to TTA (Leucine). It also harbors a second T insertion at +2036 bp within the second target site, causing a frameshift that switches the codon from CAG (Glutamine) to CTA (Leucine). *DAO2-2* exhibits a single-base (C) deletion exclusively at position +2035 bp of the second target site, driving a frameshift that converts CAG (Glutamine) to AGA (Arginine). *DAO2-3* shares the identical first-site mutation as *DAO2-1* (TAC to TTA) but displays a distinct base substitution accompanied by a 5-bp deletion at the second target site, resulting in a disruptive frameshift (Figure 1B). All identified mutations represent non-synonymous, loss-of-function variations.

### 3.2. Metabolic Profiling of Mature Brown Rice

**Figure 2:**
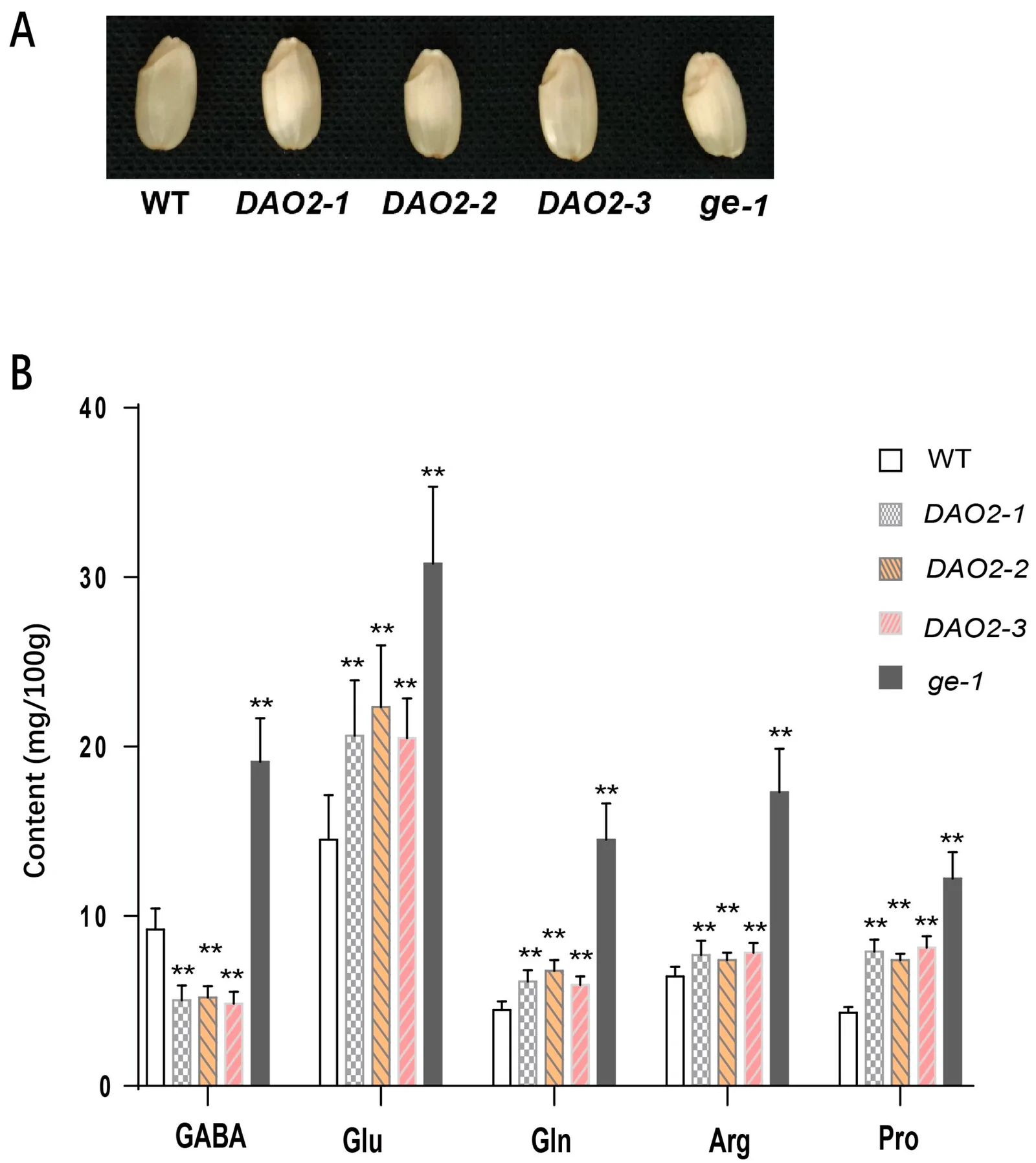
Comparison of metabolite contents in different brown rice varieties. (**A**) Phenotypes of grain appearance. (**B**) Comparison of GABA, Glu, Gln, Arg, and Pro contents in WT, *DAO2* mutants (*DAO2-1*, *DAO2-2*, and *DAO2-3*), and *ge-1* brown rice. The values are the mean ± SD (n = 10). Asterisks ** indicate a significant difference between WT and other mutant materials at *p* < 0.01.

Quantification of free amino acids in mature brown rice from the homozygous T_2_ mutant lines (*DAO2-1*, *DAO2-2*, *DAO2-3*), the giant embryo mutant *ge-1*, and the WT control revealed distinct metabolic signatures, with glutamate (Glu) consistently representing the most abundant pool across all lines (Figure 2A,B). Free GABA levels in *DAO2-1*, *DAO2-2*, and *DAO2-3* grains were 5.07 mg/100 g, 5.22 mg/100 g, and 4.85 mg/100 g, respectively. These values reflect highly significant decreases of 45.01%, 43.38%, and 47.40% relative to the WT baseline (9.22 mg/100 g). Conversely, the high-GABA control *ge-1* exhibited an elevated GABA content of 19.09 mg/100 g, marking a 107.05% increase over WT (Figure 2B).

The suppression of GABA in the *DAO2* lines was mirrored by a significant accumulation of upstream biosynthetic precursors. Glu concentrations reached 20.65 mg/100 g (*DAO2-1*), 22.35 mg/100 g (*DAO2-2*), and 20.53 mg/100 g (*DAO2-3*), compared to 14.52 mg/100 g in WT and 30.76 mg/100 g in *ge-1*. Glutamine (Gln) levels were similarly elevated in the mutants (5.96–6.79 mg/100 g) relative to WT (4.47 mg/100 g), while *ge-1* stood at 14.50 mg/100 g. Arginine (Arg) concentrations rose to 7.43–7.85 mg/100 g in the *DAO2* mutants versus 6.45 mg/100 g in WT and 17.31 mg/100 g in *ge-1*. Proline (Pro) also showed a dramatic increase, spiking from 4.30 mg/100 g in WT to 7.42–8.12 mg/100 g in the *DAO2* lines, while *ge-1* maxed at 12.21 mg/100 g (Figure 2B). These data establish that *DAO2* deficiency reduces baseline GABA levels while simultaneously triggering an over-accumulation of Glu, Gln, Arg, and Pro in mature unpolished grains. The agronomic traits of the CRISPR DAO2 transgenic lines, such as thousand-kernel weight, plant height, and heading date, were evaluated. These traits showed no significant differences compared to WT. It was demonstrated that the DAO2 gene played a significant role in the GABA accumulation process of rice seeds.

### 3.3. Spatiotemporal Metabolite Tracking During Germination

**Figure 3:**
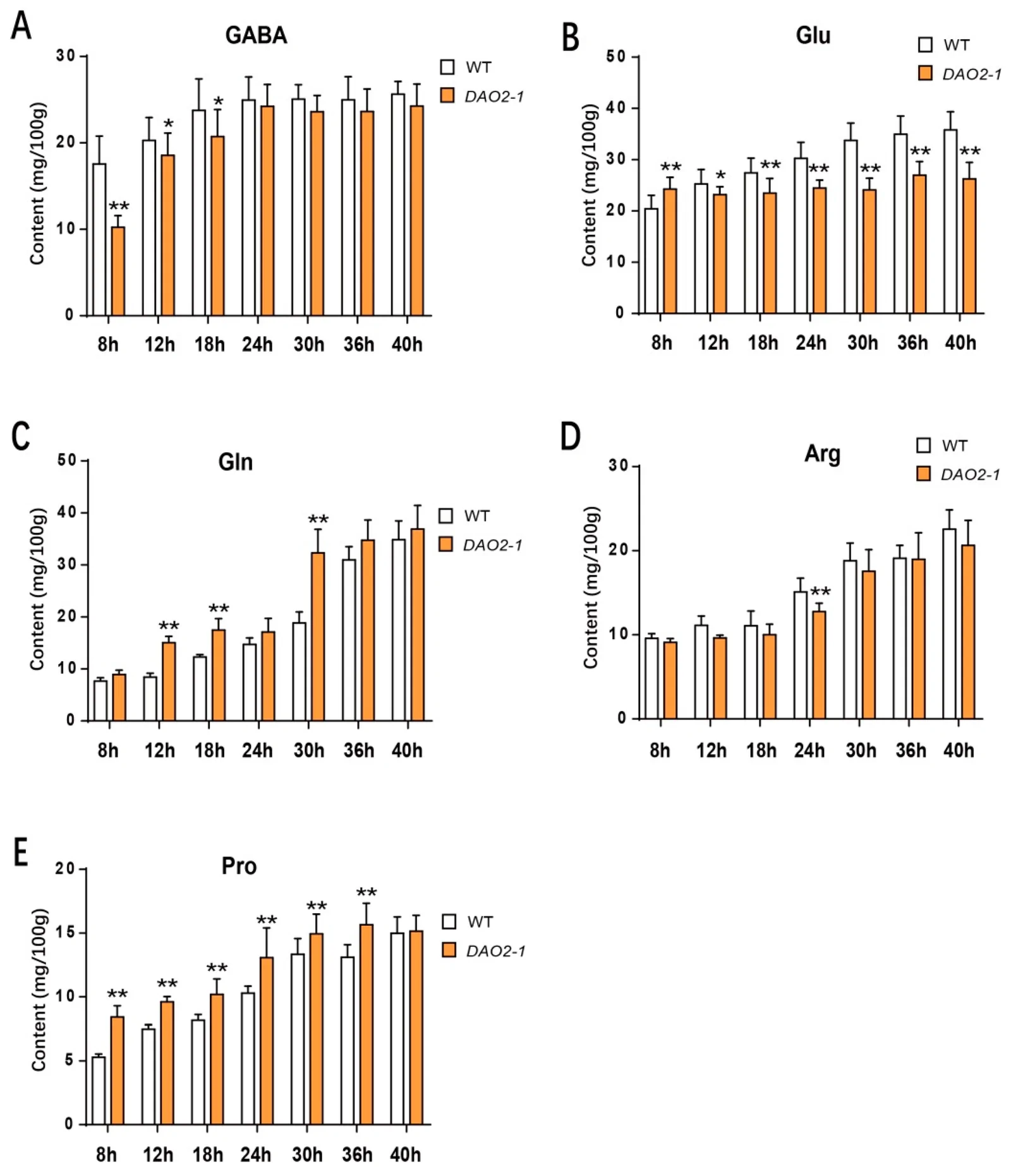
Spatiotemporal metabolic fluctuations during germination. Comparison of the accumulation profiles for GABA (**A**), Glu (**B**), Gln (**C**), Arg (**D**), and Pro (**E**) between *DAO2-1* and WT grains sampled across seven sequential incubation intervals (8 h, 12 h, 18 h, 24 h, 30 h, 36 h, and 40 h). The values are the mean ± SD (*n* = 3). Asterisks * and ** indicate significant differences in the contents of metabolites between WT and *DAO2-1* at the levels of *p* < 0.05 and *p* < 0.01, respectively.

To evaluate how *DAO2* disruption affects nitrogen mobilization during early development, the *DAO2-1* mutant and WT grains were subjected to a time-course germination study spanning 40 h (Figure 3). Both genotypes displayed a progressive rise in free GABA content that peaked at 24 h (24.24 mg/100 g for *DAO2-1*; 24.95 mg/100 g for WT) before entering a plateau phase. Over the complete germination window, GABA titers ranged from 10.23 to 24.24 mg/100 g in *DAO2-1* and from 17.55 to 25.64 mg/100 g in WT. Notably, during the early phases of germination (8, 12, and 18 h), the GABA content of *DAO2-1* was significantly lower than that of WT, though this difference dissolved as development progressed beyond 24 h (Figure 3A).

The dynamics of related metabolites also varied between the two lines. The Glu pool in *DAO2-1* remained remarkably flat throughout incubation, fluctuating narrowly between 23.19 and 26.98 mg/100 g. In contrast, WT grains demonstrated a time-dependent increase in Glu, expanding from 20.43 to 35.81 mg/100 g (Figure 3B). Consequently, the *DAO2-1* mutant showed significantly higher Glu levels than WT only at the 8-h checkpoint, with no statistical variance thereafter. Gln, Arg, and Pro contents expanded continuously over time in both lines. Gln pools climbed similarly in *DAO2-1* (8.94 to 36.90 mg/100 g) and WT (7.69 to 34.87 mg/100 g), with the mutant showing briefly elevated levels at 12 and 18 h (Figure 3C). Total Arg pools reached up to 20.64 mg/100 g in *DAO2-1* and 22.56 mg/100 g in WT, with a transiently lower level recorded in the mutant at 24 h (12.76 mg/100 g in *DAO2-1*, compared to 15.11 mg/100 g in WT) (Figure 3D). Proline dynamics showed the most striking divergence: *DAO2-1* maintained significantly higher Pro concentrations than WT across almost all time points (ranging from 8.43 to 15.15 mg/100 g, compared to 5.30 to 15.00 mg/100 g in WT), converging only at the final 40-h mark (Figure 3E). The results indicated that GABA, Glu, Gln, Arg and Pro contents of *DAO2-1* and WT germinated brown rice changed significantly at different germination times, and the trends of the changes in the contents of these five metabolites also differed greatly.

These findings clarify that while *DAO2* loss suppresses GABA synthesis during early germination, alternative metabolic pathways or feedback networks flatten these initial variations during later developmental stages, accompanied by substantial restructuring of broader amino acid pools.

### 3.4. Expression Levels of Genes of the GABA Metabolic Pathway During Germination

**Figure 4:**
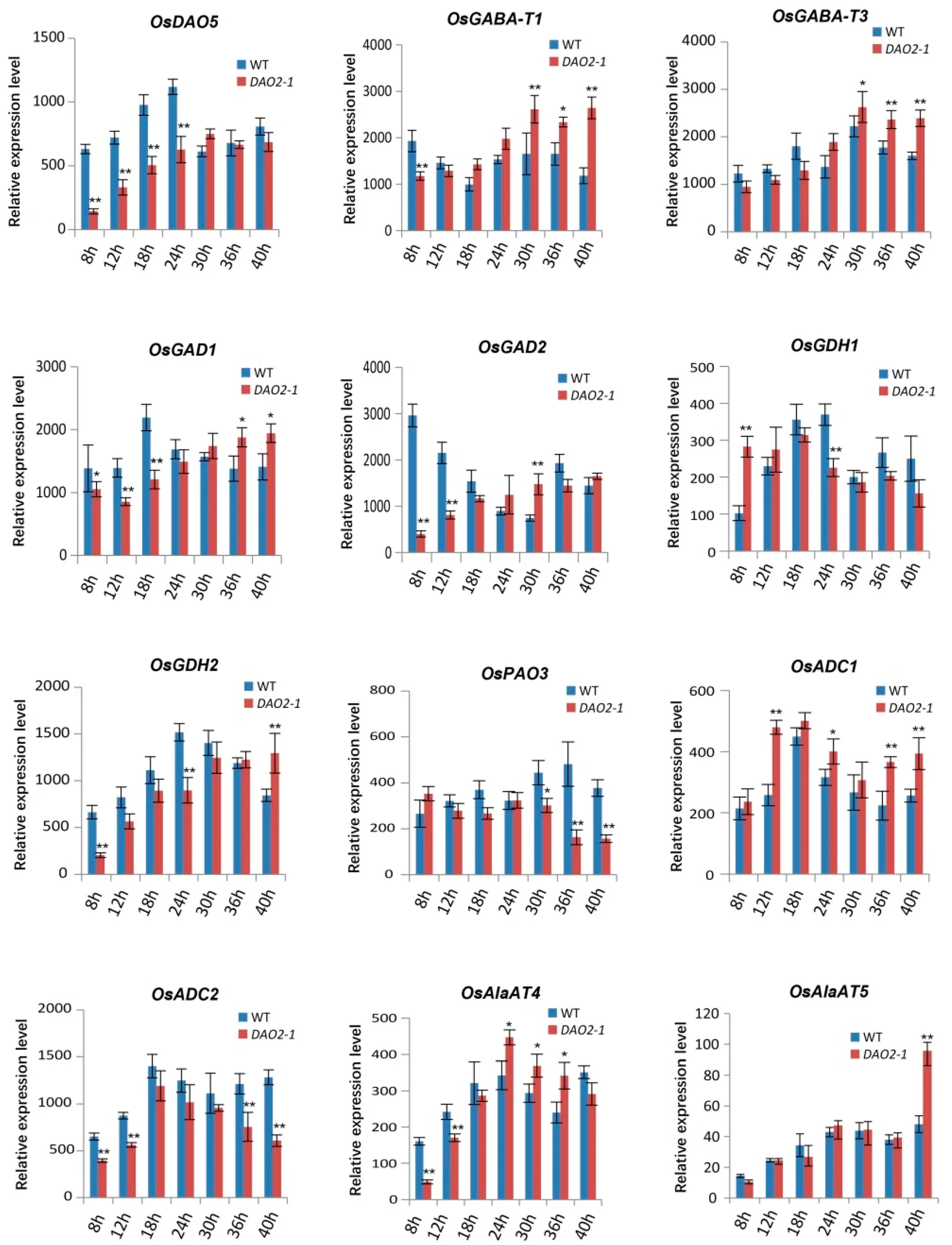
Comparison analysis of the expression levels of different genes in the *DAO2-1* mutant and the WT at different germination times. Comparison of expression levels of genes *OsDAO5*, *OsGABA-T1*, *OsGABA-T3*, *OsGAD1*, *OsGAD2*, *OsGDH1*, *OsGDH2*, *OsPAO3*, *OsADC1*, *OsADC2*, *OsAlaAT4* and *OsAlaAT5* between *DAO2-1* and WT germinated rice sampled across seven sequential incubation intervals (8 h, 12 h, 18 h, 24 h, 30 h, 36 h, and 40 h). Asterisks * and ** indicate significant differences in expression levels between WT and DAO2-1 at the *p* < 0.05 and *p* < 0.01 levels, respectively.

The distinct temporal variations observed in the five key metabolites between *DAO2-1* and WT prompted a comprehensive transcriptional analysis of the underlying metabolic network. We monitored the expression dynamics of twelve pivotal pathway genes—*OsDAO5*, *OsGABA-T1*, *OsGABA-T3*, *OsGAD1*, *OsGAD2*, *OsGDH1*, *OsGDH2*, *OsPAO3*, *OsADC1*, *OsADC2*, *OsAlaAT4*, and *OsAlaAT5*—across seven germination intervals spanning 8 to 40 h. Every investigated gene exhibited statistically significant transcript variations between genotypes at specific developmental windows, underscoring a systemic regulatory restructuring in response to *DAO2* deficiency (Figure 4).

The transcription of downstream shunt and polyamine degradation components showed clear phase-dependent shifts. Transcripts of the diamine oxidase homolog *OsDAO5* were strongly suppressed in *DAO2-1* relative to WT during the first four developmental intervals (8–24 h) before converging to wild-type levels in later stages. Conversely, genes mediating GABA catabolism and synthesis exhibited a late-induction pattern in the mutant; the gamma-aminobutyric acid transaminase genes *OsGABA-T1* and *OsGABA-T3* were significantly up-regulated in *DAO2-1* during the final phases of germination compared to WT. A closely mirrored trend was observed for the rate-limiting glutamate decarboxylase genes *OsGAD1* and *OsGAD2*, which were significantly down-regulated in early mutant germinants but rose sharply above WT levels as germination progressed.

Parallel deviations occurred within upstream amino acid and polyamine precursor pathways. The glutamate dehydrogenase genes *OsGDH1* and *OsGDH2* displayed divergent, asynchronous expression profiles; *OsGDH1* transcript abundance in *DAO2-1* exceeded WT levels at 8 h but dropped significantly below the control at 24 h; and *OsGDH2* transcript abundance in *DAO2-1* was initially suppressed at 8 h and 24 h, whereas peaking above WT at the 40-h mark. Polyamine processing was also altered; the polyamine oxidase gene *OsPAO3* maintained comparable baseline expression between genotypes initially, but was significantly down-regulated in *DAO2-1* during the final three intervals. Furthermore, the arginine decarboxylase duplicates *OsADC1* and *OsADC2* demonstrated reciprocal transcriptional responses; *OsADC1* expression was significantly elevated in the *DAO2-1* line across most time points, while *OsADC2* remained consistently repressed relative to WT. Finally, alanine transamination was modulated via *OsAlaAT4*, which was heavily suppressed at 8 h and 12 h in the mutant before surpassing WT levels during mid-to-late germination, while its homolog *OsAlaAT5* showed a highly specific, significant up-regulation in *DAO2-1* exclusively at 40 h. Taken together, these differences in expression levels indicate that *DAO2* deletion triggers a cascade of compensatory and phase-specific gene expressions across the broader GABA metabolic landscape during brown rice germination.

## 4. Discussion

### 4.1. Mutation Efficiency of CRISPR/Cas9 Multi-Target Editing at the DAO2 Locus

This study evaluated the efficacy of a dual-target CRISPR/Cas9 editing system designed to disrupt the *DAO2* locus. Sequencing revealed pronounced variations in mutation efficiency between the two targeted regions. Although site-directed mutations were successfully introduced at both positions, the structural diversity of alleles generated at the first target site was constrained compared to the highly hypermutable second site. The mutation type at the first target site was relatively small, while the mutation efficiency at the second target site was very high. Screened across 48 T_0_ transgenic individuals, 33 mutant lines were verified, all of which exhibited diverse allelic variations at the second target site, translating to a substantial single-site mutation efficiency of 68.75%. This positional variance aligns with our previous multi-target editing of the *GE* gene, where editing efficiency diverged sharply among three targets; the second and third *GE* targets yielded successful modifications while the first target remained entirely unmutated. Notably, the second *GE* target exhibited high mutational activity (79.41%), whereas the third was strictly constrained to 2.94%, potentially due to the high GC content inherent to the *GE* genomic locus, which resists standard single-guide RNA hybridization [7]. For context, Tian et al. enhanced the editing capability of CRISPR/Cas9 system in rice using the WDV-gate system [38]. In the T_0_ generation, an editing efficiency of 90.1% was achieved, and homozygous mutants were obtained [38], whereas Zhang et al. achieved a 64.3% mutation rate targeting specific site of the salinity sensitive gene *OsRR22* in japonica rice cultivar WPB106 [39]. Although our dual-target system yielded numerous single-point homozygous lines directly in the T_0_ generation, it did not induce large-scale inter-target segment deletions or insertions. The observed mutational profiles and site-specific efficiencies documented here offer a foundational benchmark for future functional genomic edits targeting *DAO2* variants across alternative cultivars.

### 4.2. Influence of DAO2 Integrity on Grain Amino Acid Pools

Metabolite analysis demonstrated that *DAO2* knockout significantly depresses baseline GABA levels while driving an over-accumulation of Glu, Gln, Arg, and Pro in mature caryopses. Plant GABA biosynthesis incorporates both cytosolic GABA shunt and mitochondrial polyamine degradation pathway [40,41], the latter featuring DAO and PAO as rate-limiting catalysts. Rice *OsDAO* is predicted to encode a 2-ketovalerate- and iron-dependent dioxygenase known to regulate pollen germination, microgametophyte development, and seed morphogenesis [11], but its precise role in endosperm amino acid partitioning has remained obscure. While giant embryo mutant lines consistently show parallel elevations in GABA, Glu, Arg [9], crude protein, and essential minerals [7], our results clarify that direct *DAO2* ablation selectively starves the total GABA pool. This systemic reduction in downstream GABA content concurrently yields a backup of upstream metabolic intermediates, most notably Glu, the primary substrate for the alternative GABA shunt. Disruption of *DAO2* within the polyamine bypass evidently down-regulates net GABA capacity in unpolished grains while inducing a highly significant compensatory storage of four functionally linked core amino acids. This sweeping alteration of the seed metabolite underscores the high interconnectivity and complexity of plant nitrogen networks. Resolving the precise flux through these competing pathways will require integrated transcriptomic and flux-based metabolomic analyses to map how *DAO2* loss reshapes the entire metabolic landscape of the developing grain.

### 4.3. Physiological Dynamics and Molecular Mechanisms of GABA Mobilization During Germination

**Figure 5:**
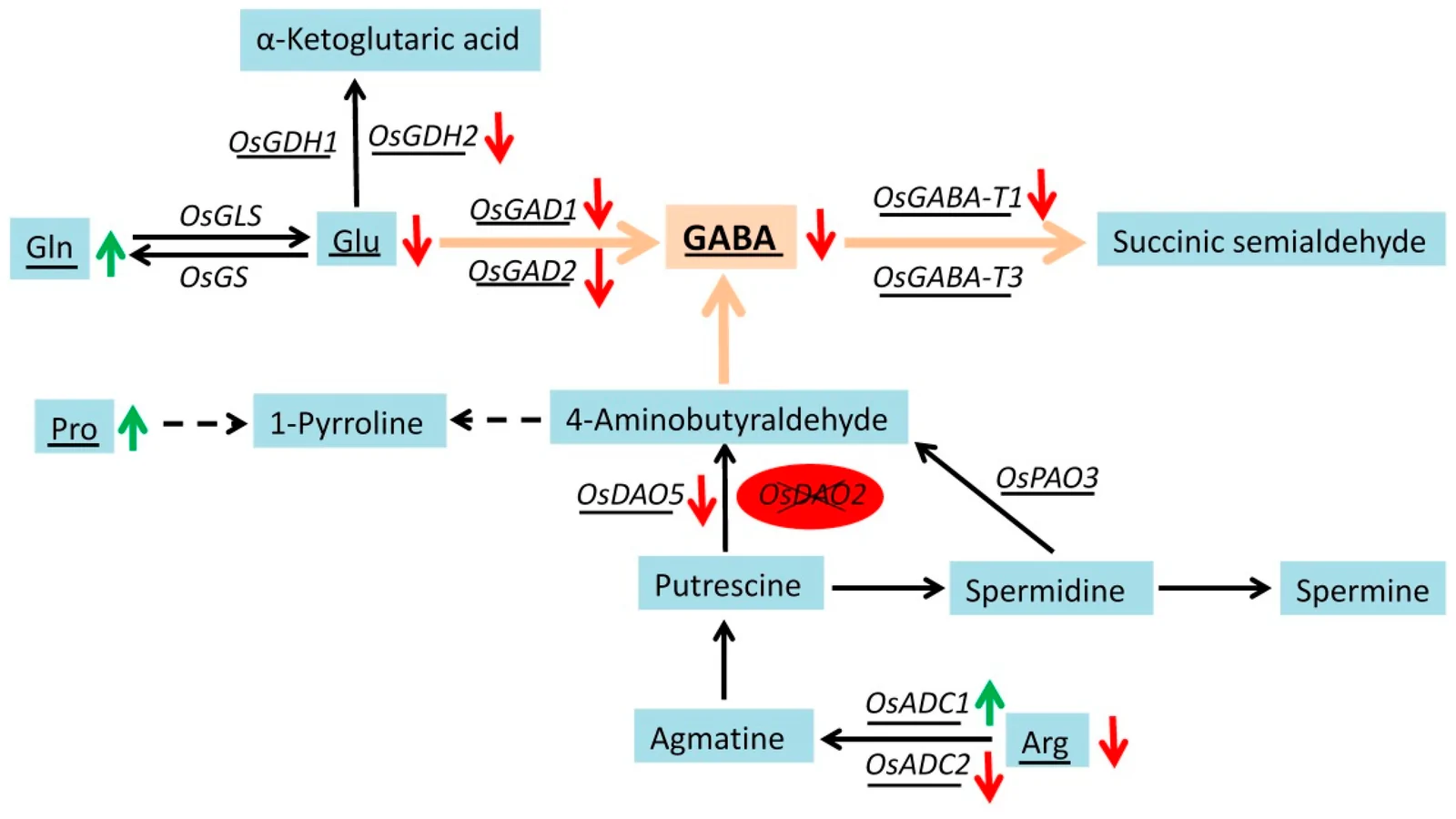
The metabolites and genes detected in this study are indicated in black underline. The upward green arrows indicate increase in metabolite content or gene expression levels in early germinants in *DAO2-1* mutant compared with those of WT, while the downward red arrows indicate decrease in metabolite content or gene expression levels in early germinants in *DAO2-1* mutant compared with those of WT. The solid arrows represent the metabolic pathways that are confirmed, while the dashed arrows represent the metabolic pathways that are questionable. *OsGLS* encodes glutaminase and *OsGS* encodes glutamine synthetase.

By monitoring germinating grain across seven key temporal windows, we confirmed that the developmental trajectory of free amino acid pools is highly genotype-dependent (Figure 3). The content changes of 5 metabolites in rice at different germination time periods were different. *DAO2-1* mutants displayed significantly lower free GABA accumulation than WT during the first three intervals (8, 12, and 18 h), a deficit that was fully resolved by 24 h, as both lines achieved maximum (reaching 24.24 mg/100 g for *DAO2-1*; 24.95 mg/100 g for WT, respectively), stable GABA titers (Figure 3). This 24-h peak matches the accumulation kinetics observed by Karladee et al. [31]. Xu et al. utilized a novel processing method in brown rice to prolong priming (PLP) and found that GABA levels in Hanyou73 (HY73) and IRAT after 10 h of germination post PLP exceeded 20 mg/100 g [42]. Saikia et al. observed the highest GABA content (20.60 mg/100 g) in Ahu rice after 24 h of steeping and 72 h of germination, which was significantly higher than that of the non-germinated control [43]. GABA content increased with the extension of germination time, and GABA content reached the maximum at 24 h of germination, and then remained relatively stable. Intriguingly, the mutant maintained significantly suppressed Glu levels relative to WT throughout the germination time course, mirroring a perfectly inverted trend in the Pro pool (Figure 3), where Pro titers in *DAO2-1* remained elevated across the first six developmental windows. Because *DAO2* loss directly shuts down polyamine-derived GABA synthesis, the rapid equalization of the total GABA pool at 24 h strongly indicates that *DAO2* deficiency triggers an up-regulation of the alternative GABA shunt to re-establish homeostatic equilibrium. Glu and Pro mainly play roles in GABA branch and the polyamine degradation pathway respectively (Figure 5). The above may be the reason for the significant decrease in Glu content in *DAO2-1* rice compared to WT germinated rice, while the change trend of Pro content is just the opposite. Given that Glu acts as the primary input for the shunt while Pro functions as a core structural component of the polyamine loop, this metabolic pivot explains the reciprocal depletion of Glu and accumulation of Pro in the mutant lines. Operationally, these findings confirm that a 24-h incubation provides the optimal processing window for maximizing GABA yields in both wild-type and engineered grains.

At the molecular level, this phenotypic shift is driven by extensive transcriptional rewiring. While Zhao et al. reported that key pathway genes, including *GADs*, *PAOs*, and *GABA-Ts* in germinated brown rice, are down-regulated in giant embryo lines compared to conventional cultivars [9], our germination time-course revealed highly dynamic, gene-specific expression profiles regulating both biosynthesis and catabolism (Figure 4 and Figure 5). In early germinants, the primary synthetic genes *OsGAD1*, *OsGAD2*, *OsGABA-T1*, *OsGDH2*, *OsDAO5*, and *OsADC2* were expressed at significantly higher levels in WT than in *DAO2-1*, while the catabolic transcripts *OsGABA-T3* remained statistically indistinguishable between the two genotypes (Figure 4 and Figure 5). Conversely, during late germination, *OsGABA-T1* and *OsGABA-T3* expression was suppressed in WT but rose significantly in the *DAO2-1* line (Figure 4). This temporal shift in the *OsGAD*/*OsGABA-T* transcriptional ratio may clarify why the mutant exhibits an initial lag in GABA accumulation before matching WT levels at later time points. Furthermore, this study provides the first transcriptional characterization of alanine aminotransferase variants (*OsAlaAT*) during brown rice germination (Figure 4). *OsAlaAT4* and *OsAlaAT5* displayed highly divergent, asynchronous expression trends, *OsAlaAT5* showed a highly specific, late-stage induction in *DAO2-1* exclusively at 40 h, whereas *OsAlaAT4* fluctuated with significant differences between genotypes across multiple germination intervals (Figure 4). Collectively, these insights demonstrate that *DAO2* plays a vital role in modulating amino acid remobilization during early development, providing a genetic and biochemical blueprint for the molecular breeding and optimized processing of GABA-fortified functional food products.

## Figures and Tables

**Table 1:** Information about the *DAO2* gene and primers used for sequencing and quantitative RT-PCR.

Gene	Locus		Chromosome		Encoding Product	
*DAO2*	*LOC_Os04g20164*		4		Amine Oxidase Precursor	
**Primers**	**Forward Primer (5′-3′)**			**Reverse Primer (5′-3′)**		
DAO2.1-F/R	GTGCTGGTCGACATGGACA			GTGAAAGTATGTTGTACGC		
DAO2.2-F/R	CGTTCCTGTGGAAACTGA			CTGTTCCCAGTACATTGA		
OsDAO5-F/R	GGATTGTTAGGAACACAAGG			GAACGATGTAACCCAAAGG		
OsGABA-T1-F/R	GTCGCCATAGAAGCCCTGAA			GTTCCAAGTATCAACCCTA		
OsGABA-T3-F/R	TGTAAGAAGCGTGGTATG			GACCTCAGCTCTGCCACT		
OsGAD1-F/R	CTGTAAAGGCTGTTGAAATG			CACCCAGTTTCCTTATTCTT		
OsGAD2-F/R	CACGACAAGCCCAACATCG			CGTAGCAGCCTTCGGTCAG		
OsGDH1-F/R	TGGGTTCAGAACATTCAGGG			TTGACACCGAGGGTGAAGG		
OsGDH2-F/R	GGGTTCAGAACATTCAAGGG			GGTTGACTCCCAGGGTGAAC		
OsPAO3-F/R	CGAGCCGATACATTACCTAG			ACTGGGATCCGCAGCTTCTC		
OsADC1-F/R	CGTCATCGACGTTGGTGGA			CCAAGCTGTATGCCACGGAC		
OsADC2-F/R	AGAAGGTTGCGACGGAGAATG			TGGTCAGCCCTTTCTTCATCA		
OsAlaAT4-F/R	TGGAAGGCGTGTCGTGTAAC			GGAGACACTGGGTGAAATCC		
OsAlaAT5-F/R	CAGCACTCCAGTTAGAGAAC			TCCATTGCCTCCTTTGCACG		
β-Actin-F/R	TGGCATCTCTCAGCACATTCC			TGCACAATGGATGGGCCAGA		

## Data Availability

The original contributions presented in this study are included in the article. Further inquiries can be directed to the corresponding author.
